# Engineering of *Nitrosomonas europaea* to express *Vitreoscilla* hemoglobin enhances oxygen uptake and conversion of ammonia to nitrite

**DOI:** 10.1186/s13568-015-0135-2

**Published:** 2015-08-01

**Authors:** Stephanie A Kunkel, Krishna R Pagilla, Benjamin C Stark

**Affiliations:** Department of Biology, Illinois Institute of Technology, Chicago, IL 60616 USA; Department of Civil, Architectural and Environmental Engineering, Illinois Institute of Technology, Chicago, IL 60616 USA

**Keywords:** Aerobic respiration, Aerobic wastewater treatment, Engineered bacteria, *Nitrosomonas europaea*, *Vitreoscilla* hemoglobin

## Abstract

*Nitrosomonas europaea* was transformed with a recombinant plasmid bearing the gene (*vgb*) encoding the hemoglobin (VHb) from the bacterium *Vitreoscilla* under control of the *N. europaeaamoC* P1 promoter. *Vgb* was maintained stably and appeared to be expressed in the transformants at VHb levels of about 0.75 nmol/g wet weight. Expression of VHb in the *N. europaea* transformants was correlated with an approximately 2 fold increase in oxygen uptake rate by whole cells at oxygen concentrations in the range of 75–100% saturation, but no change in oxygen uptake rate at oxygen concentrations below 25% saturation. VHb expression was also correlated with an increase of as much as about 30% in conversion of ammonia to nitrite by growing cells. The results suggest that engineering of key aerobic wastewater bacteria to express bacterial hemoglobins may be a useful strategy to produce species with enhanced respiratory abilities.

## Introduction

One of the key functions of the aerobic portion of conventional waste water treatment (activated sludge process) is the efficient oxidation of ammonia to nitrate, because ammonia in the effluent is toxic to aquatic species when released to the environment. As the nitrifying species are relatively intolerant of low dissolved oxygen (DO) levels, the activated sludge process is run at high DO levels, ranging from a low of 2 mg DO/L to as high as saturation at the ambient temperature (Rittmann and McCarty 2001). The variation is due to variations in the influent wastewater flow, concentrations of oxygen-demanding substances, and wastewater temperature. Furthermore, to avoid occurrences of less than 2 mg DO/L, the activated sludge process is operated at much higher DO levels than necessary. The energy required for the aeration in the activated sludge process to achieve these high DO levels is enormous, amounting to 45–75% of the energy required to run a waste water treatment plant (Rosso et al. 2008). Thus, development of nitrifying species and communities that can efficiently convert ammonia to nitrate at low DO levels [“demand-side” strategies (Arnaldos and Pagilla 2014)] could be an important aspect of energy conservation efforts.

*Nitrosomonaseuropaea* is a key member of the bacteria responsible for the first step in nitrification, conversion of ammonia to nitrite. This conversion occurs in two steps, from ammonia to hydroxylamine, and then hydroxylamine to nitrite. The first step is catalyzed by ammonia monooxygenase, in which molecular oxygen is one of the substrates. Molecular oxygen is also the terminal electron acceptor in the electron transport chain of *N.europaea* (Rittmann and McCarty 2001).

Previously we have investigated how nitrification can be achieved at low DO by adaptation of native species from activated sludge to growth at low DO in medium containing only ammonia as a source of electrons. In that study (Arnaldos et al. 2013), a community highly enriched in nitrifying species (including *N.europaea*) was obtained after 140 days that could efficiently convert ammonia to nitrate at DO levels of 0.1 mg/ml (about 1% of saturation). This community was also characterized by an increase in the levels of an as yet unidentified heme protein, which did not seem to be a peroxidase or any of the oxidases known to be involved in nitrification (Arnaldos et al. 2014).

An important group of heme proteins, bacterial hemoglobins, were initially discovered in 1986 in *Vitreoscilla*. Although the initial discovery was surprising, since then it has become apparent that bacterial hemoglobins are very common, occurring in about two thirds of all bacterial species (Vinogradov et al. 2006). There are three main classes of bacterial hemoglobins, with a number of functions, although each of these involves oxygen binding or sensing in some way (Vinogradov et al. 2006; Vinogradov and Moens 2008). It is possible, then, that the unidentified heme protein found in our earlier studies could be a hemoglobin.

The *Vitreoscilla* hemoglobin (VHb), the archetypical full-length single domain hemoglobin, is perhaps the best studied of all bacterial hemoglobins (Frey and Kallio 2003; Zhang et al. 2007; Stark et al. 2011, 2012, 2015). Among its functions are binding of oxygen (particularly at low DO) and delivery to the respiratory chain (Ramandeep et al. 2001; Park et al. 2002) to enhance oxidative phosphorylation at low oxygen concentrations, and to oxygenases, to enhance their activity (Fish et al. 2000; Lin et al. 2003). The gene (*vgb*) encoding VHb has been cloned and transformed into a variety of bacterial, fungal, and even plant species to enhance their growth and productivity, especially under low oxygen conditions (Frey and Kallio 2003; Zhang et al. 2007; Stark et al. 2011, 2015).

The documented utility of *vgb*/VHb in recombinant organisms, the ability of VHb to aid oxidative phosphorylation and oxygenases, and the possibility that one or more bacterial hemoglobins may be induced in a nitrifying community as the result of low DO adaptation, led us to investigate whether engineering of *N.europaea* to express VHb could enhance its growth, respiration and ability to convert ammonia to nitrite. This could provide support for the idea that hemoglobins which may be important in low DO functioning occur naturally in one or more nitrifiers, as well as serving as a proof of concept that engineering nitrifiers with *vgb*/VHb might be of use in development of efficient low DO activated sludge processes.

## Materials and methods

### Cell growth and maintenance

*Nitrosomonas europaea* (ATCC 19178), received from the laboratory of Dr. Luis A. Sayavedra-Soto (Oregon State University, Corvallis, Oregon), was grown in 0.5 L liquid batch cultures in 1 L Erlenmeyer flasks in the dark at 30°C with gentle agitation (~120 rpm) in 50 mM ammonium medium (Hyman and Arp 1992). Medium for transformed *N. europaea* cultures contained 25 µg/mL ampicillin.

### Construction of plasmid pSK2

**Fig. 1:**
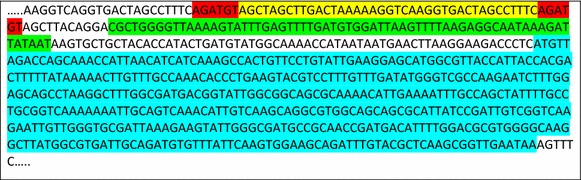
Sequence of plasmid pSK2 in the region of *vgb* and the integrated *amoC* P1 promoter. The *amoC* promoter is highlighted in *yellow*, with the flanking *Hin*dIII sites highlighted in *red*. The native *vgb* promoter region is highlighted in *green*, and the *vgb* coding sequence, beginning with the ATG start codon and ending with the TAA stop codon, is highlighted in *turquoise*.

Plasmid pUC8:16 (*vgb* cloned into the *Hin*dIII-*Sal*I sites of *E. coli* vector pUC8; Liu et al. 1994) was cleaved at its *Hin*dIII site. A synthetic sequence identical to the *N. europaeaamoC* P1 promoter (Hommes et al. 2001; Berube et al. 2007) was produced from two complementary oligonucleotides (Integrated DNA Technologies, Coralville, IA) that were 5′ phosphorylated by T4 polynucleotide kinase and annealed by heating to 95°C for 10 min followed by slow cooling to room temperature. Because of the design of the two oligonucleotides, the annealed product had 4 bp single stranded *Hin*dIII compatible overhangs at each end, which allowed sticky end ligation into the *Hin*dIII site in pUC8:16 to produce pSK2. This placed the *amoC* P1 promoter just upstream of the native *vgb* promoter (Fig. 1). The *amoC* P1 promoter was previously found to be active in the presence of ammonia (Hommes et al. 2001) and is thus a good candidate for expression of *vgb* for these studies. Because of its derivation from pUC8:16, pSK2 confers resistance to ampicillin.

### Electroporation of pSK2 into *N. europaea*

*N. europaea* cells were grown in 0.5 L cultures in the ammonia medium described above to an OD_600nm_ of ~0.1, collected by centrifugation and washed three times. The washed cell pellet was resuspended in deionized H_2_O and chilled on ice for 30 min, and 100 μL of cell suspension mixed with 1 μL (1 μg) pSK2 in a prechilled 1 mm gap electroporation cuvette. The cuvette was placed into a BTX ECM 630 Exponential Decay Wave Electroporation System (Harvard Apparatus Inc., Holliston, MA) and pulsed once at 1,200 V, 25 mF and a resistance larger than 100 ohms. Immediately after pulsing, the cells were transferred to 0.5 L prewarmed ammonia medium and grown for 24 h under non-selective conditions at 30°C and 100 rpm shaking. After 24 h ampicillin was added to a concentration of 25 μg/mL. Transformant cultures grew up after 7–14 days and they were usually maintained as liquid cultures due to the extreme difficulty in isolating colonies on plates as well the slow growth time (3–4 weeks) and fast decay on solid medium (1–2 weeks) (Sayavedra-Soto and Stein 2011).

### Plasmid purification and PCR

Plasmid preps were performed using the E.Z.N.A. Plasmid Mini Kit (Omega Bio-Tek, Norcross, GA) according to the manufacturer’s instructions. PCR analysis was also done on the plasmids in order to amplify *vgb* from (positive control) pUC8:16 (Vgb1/Vgb2 primer set) and the *amo1* promoter along with *vgb* from pSK2 (AmoCp1/Vgb2 primer set). These primers and the annealing temperatures used are listed in Table 1. The PCR program used was the following: step 1—94°C for 5 min, step 2—94°C for 30 s, step 3—temperature dependent on primer used (Table 1) for 30 s, step 4—72°C for 1 min and 15 s, step 5—72°C for 5 min and step 6—held at 4°C; amplification cycle (steps 2–4) repeated 30 times.

**Table 1:** PCR primers used for amplification of *vgb* and the *amo1* promoter

Primer	Sequence (5′–3′)	Annealing temp (°C)
Vgb1	GCG CGG AAT TCA TGT TAG ACC AGC AA	59
Vgb2	GCG CGC TCG AGT TAT TCA ACC GCT TG	59 (60)*
AmoCp1	AGC TAG CTT GAC TAA AAA GGT CAA GGT GAC TAG CCT TTC AGA TGT	62 (60)*

* Annealing temperature used for the primer pair.

### CO-difference spectra

0.5 L cultures of *N. europaea*, and *N. europaea*[pSK2] were grown for 5 days as described in “Introduction” section above, and 5 mL cultures of *E. coli* DH5α[pUC8:16] were grown in LB medium in 15 mL culture tubes at 37°C and 200 rpm. Preparation of cell extracts and CO-difference spectra determination on them (600–400 nm with a sampling interval of 1 nm) were done as described by Dikshit and Webster (1988) using a Shimadzu UV-1800 spectrophotometer.

### Nitrite assay

Nitrite production from ammonia was measured in culture samples using the spectrophotometric method of Hageman and Hucklesby (1971).

### Oxygen uptake measurements

Two 0.5 L cultures each of untransformed *N. europaea* and *N. europaea*[pSK2] cells were harvested in log phase, after approximately 2–3 days of growth and at an OD_600nm_ of approximately 0.05; the exact OD_600nm_ of each culture at the time of harvesting was recorded. The cells were pelleted by centrifugation and washed three times with deionized water, and the resulting pellet resuspended in 40 mL of the appropriate *Nitrosomonas* growth medium (ammonia medium either without antibiotics or containing 25 μg/mL ampicillin) at room temperature in a 50 mL flask. Due to the slow growth and overall low density of *Nitrosomonas europaea*, the rate of oxygen uptake in these experiments was quite slow. For this reason the OUR experiments were performed in two portions, the first measuring oxygen uptake in an initially fully saturated medium, therefore starting at 100% saturation and reading down to about 80% saturation, and the second portion starting at around 25% and finishing as close to zero as possible. The fully saturated medium was bubbled with air for 30 min prior to the start of each assay in order to ensure 100% oxygen saturation. The low oxygen medium was prepared in the same way except prior to the OUR assay the oxygen level was reduced to 25% saturation by addition of sodium dithionite.

OUR values were measured using a 5331 oxygen probe connected to a 5300A oxygen monitor (YSI, Yellow Springs, OH), which was calibrated before each assay according to the manufacturer’s instructions and then was used immediately following calibration to ensure accuracy. Immersion of the probe into the cell suspension caused the initial volume to rise, thus leaving no headspace. The conversion from per cent saturation to mg/L oxygen was determined on the basis of assay temperature and salinity.

## Results

We were able to transform *N.europaea* using two *vgb*-bearing plasmids which we had previously constructed. These included pUC8:16 (Dikshit and Webster 1988; Liu et al. 1994), in which *vgb* with its native (low oxygen responsive) promoter is cloned into the *E.coli* vector pUC8 (Vieira and Messing 1982) and pRESX-*vgb*, *vgb* cloned into the *Rhodococcus*-*E.coli* shuttle vector pRESX (van der Gieze et al. 2008), in which *vgb* transcription is driven by a *Rhodococcus* type promoter. In neither case, however, was VHb expressed, presumably because of incompatibility between promoter and host RNA polymerase.

Following this we obtained the sequence of the *amoC* P1 promoter from *N.europaea* from the literature (Hommes et al. 2001) and had it synthesized, and cloned it into our existing plasmid pUC8:16, producing plasmid pSK2. This construction maintains the native *Vitreoscilla* promoter upstream of the transcriptional start point but inserts the *N.europaea* promoter upstream of the *Vitreoscilla* promoter. The correct construct was confirmed by DNA sequencing (Fig. 1).

**Fig. 2:**
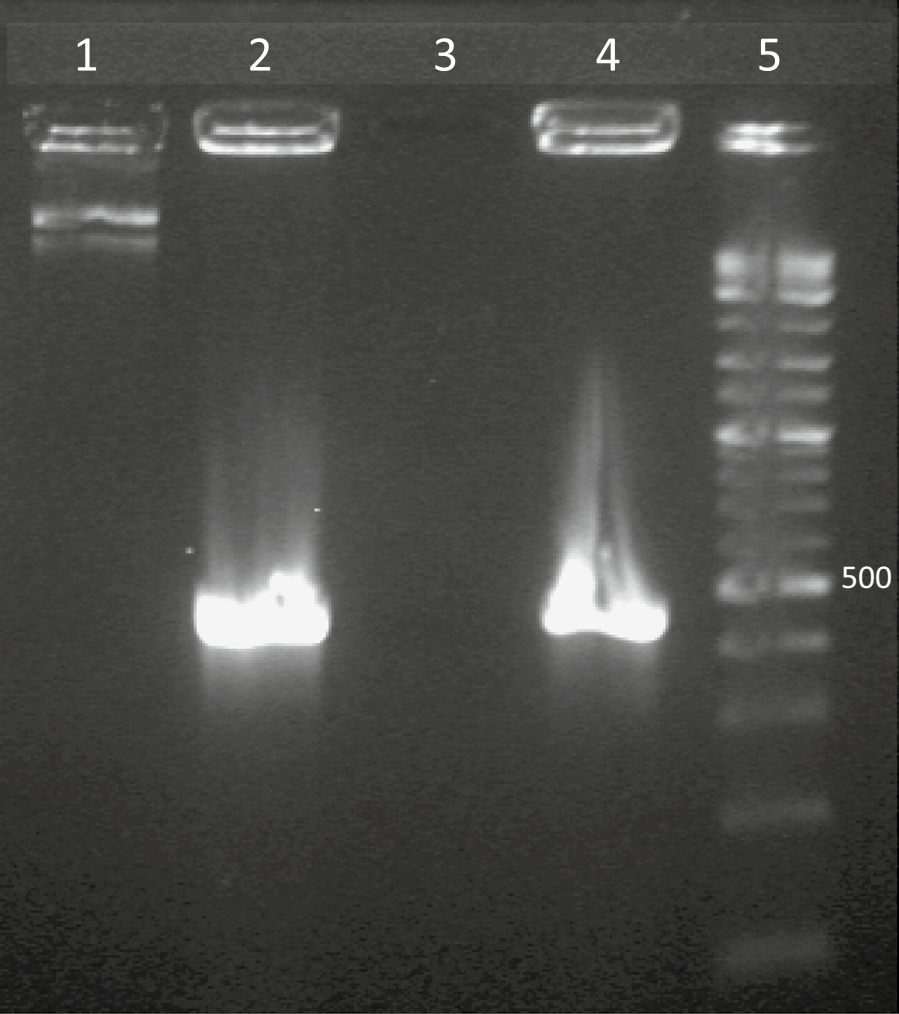
pSK2 is stably maintained in *N. europaea*[pSK2]. *Lane 1* pSK2 plasmid prep from *N. europaea* [pSK2]; *lane 2* positive control PCR of *vgb* amplicon (from pUC8:16 from *E. coli*); *lane 3* negative control PCR of *vgb* (plasmid prep from untransformed *N. europaea*); *lane 4* PCR amplicon of *vgb* from *N. europaea* [pSK2]; *lane 5* 2-log ladder (New England Biolabs; position of 500 bp fragment is noted). The amplicons in *lanes 2* and *4* are of the expected size (about 450 bp).

Transformation of *N.europaea* with pSK2 was successful and stable with antibiotic pressure (25 μg/mL ampicillin), as proven by miniprep analysis and PCR amplification of *vgb* repeated at 2 week intervals (Fig. 2). In addition, this construct allowed production of VHb in *N.europaea*, as demonstrated by CO-difference spectral analysis.

**Fig. 3:**
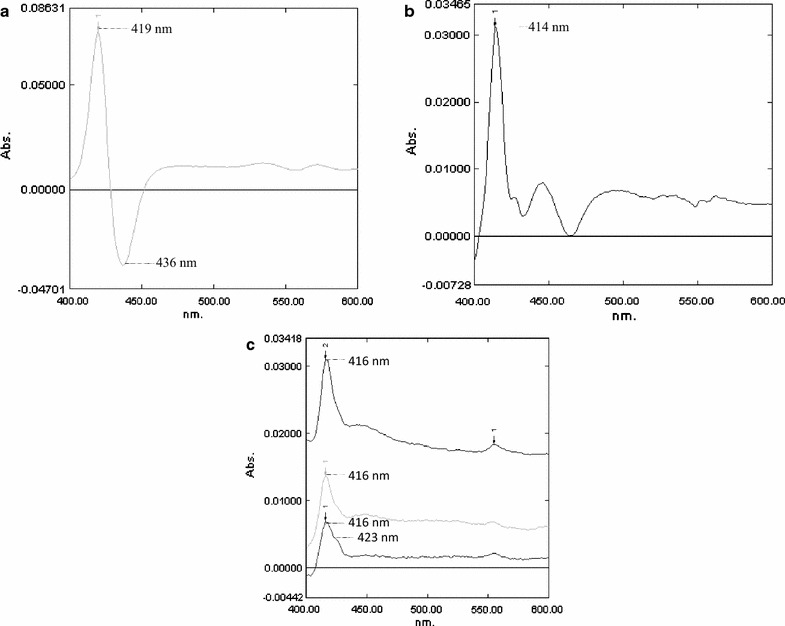
CO-difference spectral analysis. **a** CO-difference spectrum of cell extract of *E. coli* DH5α[pUC8:16] expressing VHb; peak and trough at 419 and 436 nm, respectively, characteristic of VHb are indicated. **b** CO-difference spectrum of cell extract of untransformed *N. europaea*; characteristic peak at 414 nm is indicated. **c** CO-difference spectra of cell extracts from three individual isolates of *N. europaea* transformed with *vgb* on plasmid pSK2; peak at 416 nm and shoulder at 423 nm are indicated.

VHb has a characteristic CO-difference spectrum with a peak at 419 nm and trough at 436 nm (Dikshit and Webster 1988; Fig. 3a). A CO-difference spectrum of a whole cell extract of untransformed *N.europaea* has several peaks, the most prominent of which is at 414 nm (Arnaldos et al. 2013; Fig. 3b). The identity of this peak is as yet unknown. CO-difference spectra of eight individual *N.europaea*/pSK2 transformants showed the 414 nm peak shifted from 414 to 416 nm with a shoulder at about 423 nm (three examples shown in Fig. 3c). The shift and shoulder are consistent with an overlap or combination of the 414 nm peak and the characteristic 419 nm VHb peak, the 423 shoulder presumably being part of the VHb signal. Although it is difficult to quantify the level of VHb from the height of the 423 shoulder, the average of calculations from three independent *N. europaea*[pSK2] spectra yielded a value of about 0.75 nmol/g wet weight of cells.

**Fig. 4:**
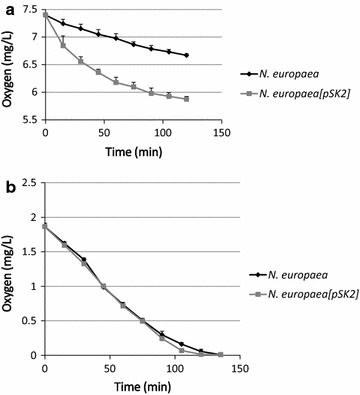
Oxygen uptake measurements for *N. europaea*[pSK2] (*gray squares* and *gray line*) and untransformed *N. europaea* (*black diamonds* and *black line*). **a** Measurements made starting with DO at 100% saturation. **b** Measurements made starting with DO at 25% saturation. All points are averages of five independent experiments; for every experiment at both initial oxygen concentrations and for both strains, the same mass of cells (0.06 OD_600nm_) was tested. *Error bars* indicate standard deviations (which in some cases are smaller than the diameter of the *symbols*).

The transformant was compared with the untransformed control strain regarding respiration (SOUR) at both high oxygen concentration (starting at 100% saturation) as well as low oxygen concentration (starting at 25% saturation) (Fig. 4). Starting at 100% DO the SOUR of the transformant was substantially greater than that of the untransformed strain (Fig. 4a), but starting at lower DO the SOUR of the two strains was essentially identical (Fig. 4b).

**Fig. 5:**
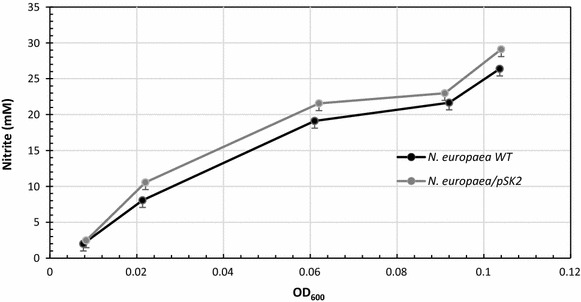
Ammonia to nitrite conversion as a function of cell mass (OD_600 nm_) for growing cultures of *N. europaea*[pSK2] and untransformed *N. europaea*. Each point is the average of 10 independent experiments; *error bars* indicate standard deviations.

The transformant was also compared with the untransformed control strain regarding production of nitrite from ammonia during growth in ammonia medium (Fig. 5). These experiments were conducted from OD_600 nm_’s of about 0.008–0.104 (corresponding to growth during 5 days following inoculation). Earlier in the growth phase (OD_600nm_ up to about 0.02, or about 2 days) the expression of VHb was correlated with an approximately 30% increase in nitrite conversion per unit of cell mass compared to the untransformed strain; the transformant’s advantage decreased gradually to only about 6% by OD_600nm_ of about 0.10 (about 5 days of growth).

## Discussion

Plasmid pSK2 is a derivative of the Messing vector pUC8 and thus has a ColEI type replication origin, native for *E. coli*. We have not been able to find any previous studies in which a ColEI replication origin is recognized in *N. europaea*. One mechanism of ColEI replication initiation requires both host RNA polymerase and RNase H, and *N.europaea* does have the latter enzyme (Chain et al. 2003); RNase H independent mechanisms for initiation of ColE1 replication also exist (Dasgupta et al. 1987). In any case, we were able to transform *N. europaea* with pSK2 many times, and frequent plasmid preparations and PCR amplification of *vgb* from these transformants confirmed that pSK2, once transformed into *N. europaea*, was stably maintained.

The level of VHb measured in *N. europaea*[pSK2] is much lower than the induced level in native *Vitreoscilla* (30 nmol/g wet weight of cells; Dikshit and Webster 1988) or in recombinant *E. coli* expressing VHb from the native *vgb* promoter (hundreds of nmol/g wet weight of cells; Dikshit and Webster 1988). Levels of VHb in a variety of other heterologous hosts are substantially lower (Patel et al. 2000; Liu et al. 1995; Dogan et al. 2006). The lowest level which we have seen that is correlated with an apparent positive effect is 4 nmol/g wet weight (in *Gordonia amarae*; Dogan et al. 2006). The results presented here suggest that an even lower VHb level can provide benefits to a recombinant bacterium.

The dissociation constant, K_d_ for oxygen of VHb when expressed in either *E. coli* or *Vitreoscilla* is 6 μM (approximately 2.3% of oxygen saturation at room temperature), and this is the DO range at which one might expect the presence of VHb to have the greatest effect on oxygen uptake rates (Webster 1987). The opposite, however, was observed. There was a substantial increase in SOUR correlated with the presence of VHb from 100% to about 80% oxygen saturation (about 231 to about 181 μM), but no difference in SOUR between the transformed and untransformed strains from 25 to 0% saturation (about 58–0 μM). The reasons for this are unknown, but it is known that several of the VHb functions depend upon its interaction with “partner proteins” (Stark et al. 2012, 2015), and it may be that the partners with which it might interact in *N. europaea* alter its oxygen binding properties.

In any event, the increased SOUR coincident with apparent VHb expression may be related to the enhancement of ammonia to nitrite conversion seen for *N. europaea*. This enhancement appears to be such that the conversion per unit of cell mass is greater rather than being due to an increase in cell mass. Two mechanisms by which this might occur are enhanced delivery of oxygen to the end of the respiratory chain (Ramandeep et al. 2001; Park et al. 2002) to enhance ATP production and thus general cell robustness (for example, greater levels of ammonia monooxygenase) or delivery directly to ammonia monooxygenase to enhance its activity. The latter mechanism appears to be the case for at least one other oxygenase (Fish et al. 2000; Lin et al. 2003).

Even if the advantages provided by the *vgb*-expressing *N. europaea* strain or other engineered VHb-expressing bacteria are great enough to be of practical usefulness, it is probably not reasonable to augment sludge in an actual treatment plant with such cells. Bacteria that express hemoglobins (full-length single domain (VHb-like) and truncated) that are known or thought to be involved in oxygen binding and delivery (Hill et al. 1996; Thorsteinsson et al. 1999; Pathania et al. 2002; Wainwright et al. 2005; Stark et al. 2011, 2012) are known to occur naturally in a number of normal sludge bacteria. The work described here suggests that the expression of VHb in a key activated sludge species may, in fact, enhance sludge performance in regard to oxygen utilization. This then suggests that enhancement of the growth of the natural hemoglobin-expressing sludge flora, or enhancement of horizontal transfer of the hemoglobin genes to other species within the sludge, may be successful demand-side strategies regarding sludge aeration. Such strategies might impact both nitrification and removal of organic carbon from wastewater.
